# Insight into the etiology of Alzheimer's disease from GLP‐1R knockout mice: Commentary on “Associations of semaglutide with first‐time diagnosis of Alzheimer's disease in patients with type 2 diabetes”

**DOI:** 10.1002/alz.70033

**Published:** 2025-02-24

**Authors:** Garth J. Thompson

**Affiliations:** ^1^ iHuman Institute ShanghaiTech University Shanghai China

1

*Alzheimer's & Dementia* recently published a study by Wang et al.,^1^ which demonstrated a link between semaglutide, an agonist for the glucagon‐like peptide‐1 receptor (GLP‐1R), and reduced diagnosis of Alzheimer's disease (AD). The authors used statistical methods to simulate a clinical trial (e.g., propensity score matching) and discovered that the ratio of a first‐time diagnosis of AD compared to no diagnosis was substantially lower in patients receiving the GLP‐1R agonist semaglutide for diabetes. A significant difference in this ratio held even when considering factors such as sex, age, and obesity status.

The etiology of AD is complex. In addition to amyloid beta plaques and tau tangles, AD symptoms also include changes to brain function, oxygen and glucose metabolism, and the balance of these factors across the entire brain.^2^, ^3^ GLP‐1R has high concentrations across much of the central nervous system.^4^ While semaglutide does not pass the blood–brain barrier, it can pass through tanycytes lining ventricle walls and thus enter several brain regions protected by the blood–brain barrier.^4^ Therefore, a neurological mechanism for the protective effect of GLP‐1R agonism in preventing AD seems likely. However, the functional and metabolic effects of GLP‐1R at the scale of the entire brain remain poorly understood.

My group recently published a study of GLP‐1R in the context of diabetes,^5^ but I had not considered that work in the context of AD until I read the recent study by Wang et al.^1^ While unexpected, this context was intriguing, as my group's work may help explain the connection between GLP‐1R and AD.^5^ In our study, we compared mice lacking the gene for GLP‐1R (*GLP1R*) to matched wild‐type mice. In both groups, we investigated the whole brain's response to exogenous glucose using positron emission tomography and deuterium magnetic resonance spectroscopy. We also examined the correlation of functional neural signals between different brain regions, or “functional connectivity,” measured with functional magnetic resonance imaging (fMRI). In the GLP‐1R–deficient mice, we observed dramatic differences from the wild‐type mice. The uptake of glucose (Figure 1A) and the rate of glucose metabolism were reduced in these mice, and this was correlated with a decrease both in functional connectivity (Figure 1B), and a decrease of band‐limited signal power in brain networks (Figure 1C).^5^

**FIGURE 1:**
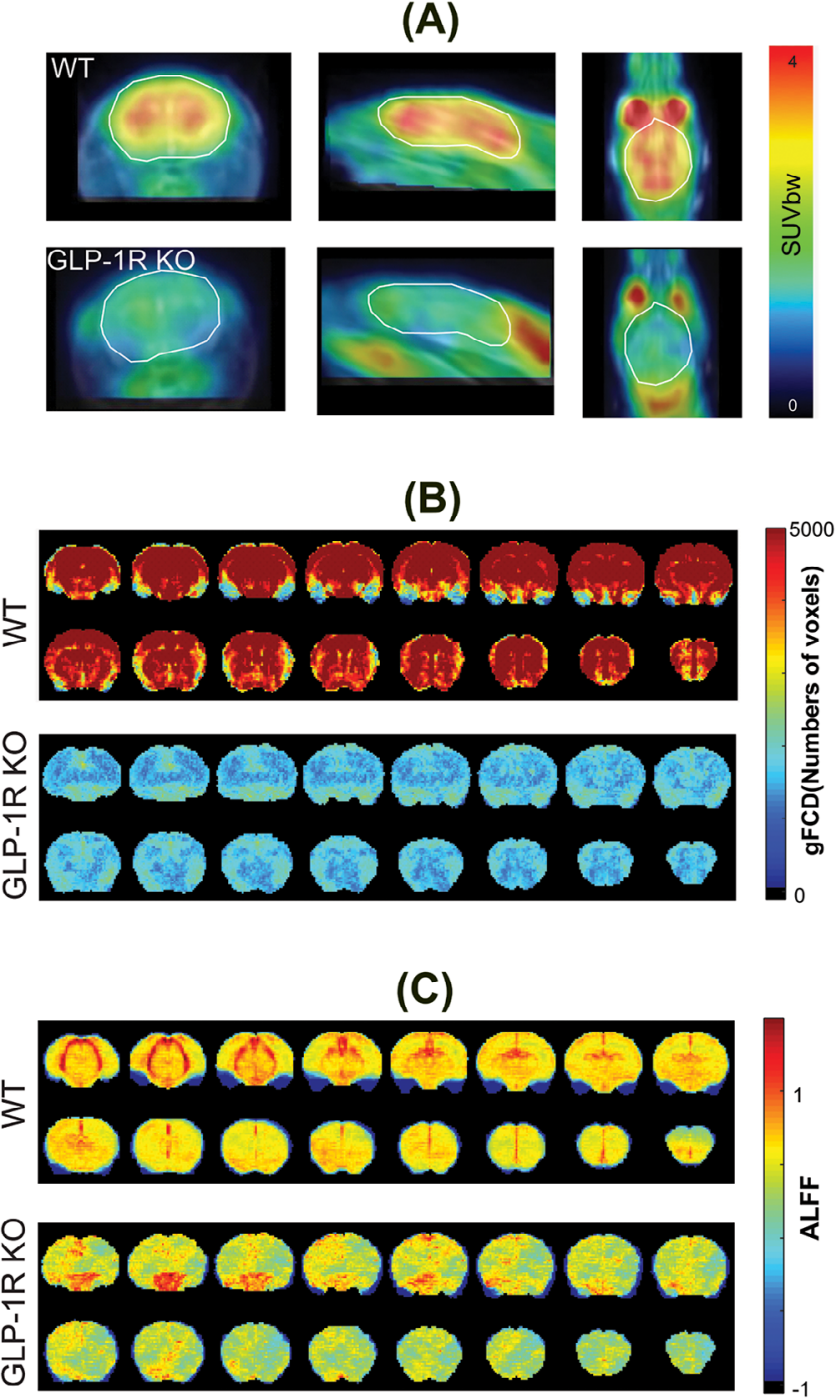
Difference between wild‐type (WT) mice and mice with double knockout of the gene *GLP1R* (GLP‐1R KO) illustrating changes in brain metabolism and functional connectivity. (A) Representative standardized uptake value corrected for body weight (SUVbw) images from coronal, sagittal, and axial views of WT and GLP‐1R KO mice taken 45–60 min after injection of [^18^F]‐Fluorodeoxyglucose. The GLP‐1R KO mouse has lower glucose uptake across the whole brain. (B) Global functional connectivity density (gFCD) maps and (C) amplitude of low‐frequency fluctuations (ALFF) maps, coronal images, 16 slices shown, an average of all mice in each group, *N* = 5 for GLP‐1R KO mice, *N* = 11 for WT mice. The average from GLP‐1R KO mice is lower across the whole brain for both gFCD and ALFF. This figure and caption were adapted with changes from Li and Fang, et al.^5^ used with permission as per the Creative Commons Attribution 4.0 International License http://creativecommons.org/licenses/by/4.0/.

Intriguingly, a similar dramatic reduction of glucose uptake and glucose metabolic rate has also been observed in human patients with AD.^3^ Comparing this to functional connectivity is difficult as most human studies perform “global signal regression.” In this type of pre‐processing, the “global signal” is calculated as the average signal from all voxels. Then, a model is fit to the global signal, and only the residuals from this model are retained. Unfortunately, global signal regression eliminates the functional–metabolic coupling that would be otherwise observed.^6^ However, a study done before the global signal regression step was common indicated reduced functional connectivity in AD,^2^ and the global signal itself has been shown to have reduced average power in AD.^7^ Taken together, these studies of glucose metabolism and fMRI in AD also strongly suggest correlated reductions in glucose metabolism and functional connectivity.

Thus, as GLP‐1R deficiency and AD both demonstrate a correlated reduction of glucose metabolism and functional connectivity, and as GLP‐1R agonists decrease the risk of AD, this strongly suggests a link between GLP‐1R function across the entire brain and the etiology of AD. The long half‐life of semaglutide (165 hours, compared to e.g., 30 minutes for beinaglutide)^8^ may better create a chronic positive change in GLP‐1R signaling compared to other GLP‐1R agonists,^1^ thus producing the opposite effect of full genetic knockout of *GLP1R*.^5^

In addition to chronic conditions such as AD and lack of GLP‐1R, global metabolic shifts which correlate with a global shift in functional connectivity also occur in the brain over relatively short time scales; this has been observed for awake versus anesthesia in rodents^9^, ^10^ and for eyes open versus eyes closed in awake humans.^6^ It is currently unknown what mechanism lies behind these linked shifts in global brain metabolism and global functional connectivity. However, the comparison between GLP‐1R–deficient mice and human AD patients not treated with semaglutide may indicate a relationship between AD and global regulation of metabotropic receptors such as GLP‐1R. If scientists are able to solve this mystery, it may help clinicians predict who will get AD, and why.

## CONFLICT OF INTEREST STATEMENT

The author declares no conflicts of interest. Author disclosures are available in the supporting information.

## FUNDING INFORMATION

The author thanks Basavaraju Ganganna Sanganahalli and Wendy Hu for reading this letter prior to submission. The author was supported by ShanghaiTech University, the Shanghai Municipal Government, and the National Natural Science Foundation of China (Grant 81950410637).

## Supporting information



Supporting Information
